# Effect of nonsurgical periodontal therapy on haematological parameters in grades B and C periodontitis: an exploratory analysis

**DOI:** 10.1007/s00784-020-03292-7

**Published:** 2020-05-08

**Authors:** Peter Eickholz, Mario Schröder, Anne Asendorf, Beate Schacher, Gerhard M. Oremek, Frank Kaiser, Martin Wohlfeil, Luigi Nibali

**Affiliations:** 1 Department of Periodontology, Center for Dentistry and Oral Medicine (Carolinum), Johann Wolfgang Goethe-University Frankfurt/Main, Theodor-Stern-Kai 7 (Haus 29), 60596 Frankfurt am Main, Germany; 2 Department of Laboratory Medicine, Centre for Internal Medicine, Hospital of the Johann Wolfgang Goethe-University Frankfurt/Main, Theodor-Stern-Kai 7, 60590 Frankfurt, Germany; 3 Centre for Host-Microbiome Interactions, Kings College London, Guy’s Hospital, Great Maze Pond, SE1 9RT London, UK

**Keywords:** Haematocrit, Mean erythrocyte volume, Number of platelets, Heat shock protein 27, Periodontitis grades B and C

## Abstract

**Aim:**

Assessment of the effect of nonsurgical periodontal therapy on haematological parameters in patients with grades B (BP) and C periodontitis (CP).

**Methods:**

Eight BP and 46 CP patients received full-mouth periodontal debridement within 48 h, if positive for *Aggregatibacter actinomycetemcomitans* with adjunctive systemic antibiotics (4 BP, 17 CP). Clinical data were collected prior and 12 weeks after periodontal therapy. Blood was sampled prior to and 1 day as well as 6 and 12 weeks after the first SD visit. Erythrocyte count, haemoglobin value, haematocrit (HCT), mean erythrocyte volume (MCV), mean corpuscular haemoglobin (MCH), MCH concentration (MCHC), platelets (PLT) and heat shock protein 27 (Hsp27) were assessed.

**Results:**

Both groups showed significant clinical improvement (*p* < 0.05). Using univariate analysis, MCV was noticeably lower in CP than BP at all examinations, HCT only at baseline. For CP, MCHC was noticeably higher 12 weeks after SD than at baseline and 1 day (*p* ≤ 0.005) and Hsp27 increased noticeably at 1 day (*p* < 0.05). Repeated measures analysis of variance revealed African origin to be associated with lower MCV and female sex with lower MCHC.

**Conclusion:**

Based on multivariate analysis, periodontal diagnosis (BP/CP) was not associated with haematological parameters measured in this study or serum Hsp27. In CP, nonsurgical periodontal therapy improved MCHC 12 weeks after SD. Also in CP Hsp27 was increased 1 day after SD.

## Introduction

Depending on individual predisposition and modifying factors, periodontitis is initiated at different ages and progresses at different speeds in different patients. The 1999 Classification of Periodontal Diseases considered this by distinguishing between chronic (ChP) and aggressive periodontitis (AgP) [1]. The current classification represents different rates of progression by assigning different grades (A, B, C); some characteristics of AgP survive as molar-incisor-pattern and case phenotype [2]. AgP and periodontitis grade C exhibit more rapid progression. This may be due to a hyperinflammatory phenotype that may be also detected in the serum by a higher inflammatory burden (i.e. C-reactive protein [CRP] and neutrophil elastase [NE]) as shown for AgP [3–5].

Extracellular cell stress proteins have the capacity to control inflammatory response and as such have been proposed as useful biomarkers e.g. heat shock protein 27 (Hsp27) to indicate anti-inflammatory activity [6]. The myeloid cell–modulating cell stress protein Hsp27 has been described to exert anti-inflammatory activities [7, 8]. Patients suffering from AgP have been shown to exhibit lower serum levels of Hsp27 than ChP patients and periodontally healthy controls [9]. Furthermore, periodontal treatment has previously been shown to increase the plasma level of Hsp10 [10].

Patients suffering from severe untreated periodontal disease frequently experience bacteraemia after tooth brushing, flossing and chewing [11]. Frequent bacteraemia and systemic spill of proinflammatory cytokines [12] from periodontal pockets result in the release of leukocyte elastase and acute phase proteins (e.g. CRP). Systemic inflammation is also associated with effects on red blood cells, possibly mediated by the effect of IL-6 and hepcidin and/or by reduced erythropoiesis. This phenomenon has been named ‘anaemia of chronic inflammation’ since it tends to occur in chronic inflammatory conditions [13]. In fact, reductions in haemoglobin levels, circulating red blood cells, haematocrit and mean corpuscular haemoglobin are suspected to be associated with both aggressive and chronic periodontitis [14, 15]. However, no studies, to our knowledge, have assessed differences in these parameters between BP and CP cases.

This is an exploratory analysis of a prospective cohort study originally aimed on inflammatory serum parameters in chronic and aggressive periodontitis [3, 4]. The aim of this exploratory analysis therefore was to compare the effect of nonsurgical periodontal therapy (subgingival debridement: SD) with haematological parameters and heat shock proteins in patients with grades B (BP) and C (CP) periodontitis.

## Material and methods

This is the exploratory analysis of data of a prospective study on the effect of nonsurgical periodontal therapy on serum inflammatory parameters. Clinical examinations and therapy have been described in detail before [4]. Thus, only a brief description is provided in the following. Sixty-six patients with untreated severe periodontal disease (31 generalized severe ChP; 35 AgP) were recruited at the Department of Periodontology of the Center for Dentisty and Oral Medicine (Carolinum), Johann Wolfgang Goethe-University Frankfurt/Main.

### Inclusion criteria

- ≥ 16 years of age
- ≥ 20 remaining teeth
- written informed consent

### Aggressive periodontitis

- Patient is clinically healthy i.e. he or she does not suffer from systemic diseases predisposing to periodontitis (e.g. diabetes mellitus)
- Probing pocket depths (PPD) ≥ 3.6 mm at more than 30% of sites [4]. According to the Periodontal Screening and Recording (PSR) index [16] and the guidelines for treatment of statutorily insured patients in Germany (Bundesausschuss der Zahnärzte und Krankenkassen 2006), a PPD of 3.5 mm is the threshold for periodontal disease and thus requirement of therapy. The Florida Probe allows measurements to the nearest 0.2 mm. Thus, PPD ≥ 3.6 mm were used as threshold for periodontal disease.
- Radiographic bone loss ≥ 50% at a minimum of 2 separate teeth
- Age at time of diagnosis ≤ 35 years (severe periodontitis below age up to 35 years is a rough threshold to identify rapid destruction in AgP) [3, 17]
- Age at time of recruitment ≤ 37 years of age [3]

### Generalized severe chronic periodontitis

- PPD ≥ 3.6 mm and probing vertical attachment loss (PAL-V) ≥ 5 mm at more than 30% of sites
- PPD ≥ 7 mm at a minimum of 4 sites (to provide a minimum of deep pockets in each patient)
- > 35 years of age

### Exclusion criteria

- Patients that may require preventive use of systemic antibiotics for measurements that may cause transitory bacteraemia (e.g. pocket probing)
- Self-reported chronic disease influencing the serum CRP level (e.g. rheumatoid arthritis, Crohn’s disease or ulcerative colitis)
- Self-reported infectious disease within the last 8 weeks before examination (history of fever)
- Any clinically assessed chronic dermal or mucosal inflammatory condition (e.g. lichen planus)
- Nonsurgical or surgical periodontal treatment within the last 24 months before examination
- Systemic or topical subgingival antibiotics within the last 8 weeks before examination

Current body weight and height as well as current and past cigarette smoking habits were recorded (self-report). Patients who reported smoking or had quit smoking for less than 5 years were classified as smokers [18]. Additionally, ethnic origin was recorded [3]. The study complied with the rules of the Declaration of Helsinki and was approved by the Institutional Review Board for Human Studies of the Medical Faculty of the Goethe-University Frankfurt/Main (Application# 188/06). All participating individuals were informed on risks and benefits as well as the procedures of the study and gave written informed consent.

### Clinical examination

Clinical examinations are reported in detail elsewhere [3].

Gingival Bleeding Index (GBI) [19] and Plaque Control Record (PCR) [20] were assessed at 6 sites per tooth (mesiobuccal, buccal, distobuccal, mesiooral, oral, distooral) at baseline, and 6 and 12 weeks after subgingival debridement (SD). Probing parameters were scored immediately before the first session of SD, and 12 weeks later. Probing pocket depth (PPD) (standard probe) and relative vertical probing attachment level (RAL-V) (disk probe) were measured to the nearest 0.2 mm using an electronic probe (Florida Probe, Version 3.2, Gainesville, USA). Bleeding on probing (BOP) was assessed 30 s after probing. Recession was measured to the nearest 0.5 mm using a manual periodontal probe (PCPUNC 15, Hu-Friedy, Chicago, USA) from the cemento-enamel junction (CEJ) to the gingival margin. Probing vertical attachment loss (PAL-V) was calculated as a sum of PPD and recession. If the CEJ was located apical to the gingival margin, PAL-V was calculated as the PPD minus the distance from the gingival margin to the CEJ. All measurements were performed by one examiner (MW).

### Reclassification

Using the baseline interproximal PAL scores and number of teeth lost due to periodontitis, each patient was assigned to a stage [2]. Missing 3rd molars were never considered as lost due to periodontal reasons. For each patient, the percentage of teeth assigned to stage III was documented.

Using radiographs obtained at baseline (primary criteria) as well as modifying factors (smoking, diabetes mellitus), each patient was assigned to a grade. The radiographs were viewed on a screen (Universial Viewer, Dentsply Rinn®, York, USA) in a darkened room by an experienced periodontologist (PE). At the tooth with most severe bone loss, the distances from the cemento-enamel junction (CEJ) to the most apical extension of bone loss (BD) and to the tip of the root were measured to the next 1.0 mm with a periodontal probe (PCPUNC15, Hu-Friedy, Chicago, USA). By dividing the distance from CEJ to BD by the distance CEJ to root tip, bone loss relative to root length was calculated. Division of relative bone loss by patients’ age provided the bone loss age coefficient. Patients with bone loss age coefficient > 1 were assigned a grade C [2].

### Blood samples

Twenty millilitres of blood were sampled from an arm vein at the following times: immediately prior to baseline scoring of probing parameters, 1 day later immediately prior to the 2nd session of SD and 6 and 12 weeks after SD. Patients were instructed not to be physically active before the blood sample. Intake of food was not standardized. Immediately after obtaining blood samples, they were transferred to the Department of Laboratory Medicine for analysis. Using the Humacount 5 haematology analyzer (Human Gesellschaft für Biochemica und Diagnostica mbH, Wiesbaden, Germany), erythrocyte count, haemoglobin value (HGB), haematocrit (HCT), mean erythrocyte volume (MCV), mean corpuscular haemoglobin (MCH), mean corpuscular haemoglobin concentration (MCHC) and number of platelets (PLT) were analyzed at the Department of Laboratory Medicine, Centre for Internal Medicine, Hospital of the Johann Wolfgang Goethe-University Frankfurt/Main. The haematology analyzer uses the volumetric impedance method.

For Hsp27 analysis, serum samples were sent to the UCL Eastman Dental Institute. Hsp27 in serum was measured using an in-house-developed two-site enzyme-linked immunosorbent assay (ELISA), as described before [9]. Briefly, 96-well plates (Immuno MaxiSorp, Nunc) were coated with a mouse monoclonal anti-HSP27 antibody (clone 2A5, ATGen) at 1 μg/ml in coating buffer (50 mM carbonate-bicarbonate buffer, pH 9.6) overnight at 4 °C. Plates were washed with wash buffer (PBS, 0.05% Tween-20), and non-specific binding sites were blocked by incubation with 2% (w/v) bovine serum albumin (BSA Chromatopur Immunoassay Grade, MP Biomedicals, Irvine, USA) in PBS for 1 h at room temperature. After washing, human recombinant Hsp27 protein (ATgen; 0–200 ng/ml) or dilutions (PBS) of serum samples and controls were added and incubated for 2 h at room temperature. Plates were washed extensively with wash buffer and incubated for 2 h at room temperature with a goat polyclonal anti-Hsp27 antibody (C-20, Santa Cruz; 1 μg/ml in 0.5% BSA/PBS). After washing, bound goat anti-hsp27 antibody was detected by incubation with HRP-conjugated donkey polyclonal antigoat IgG (Santa Cruz ssc-2304, mouse and human adsorbed, 40 ng/ml in 0.5% BSA in wash buffer) at room temperature for 45 min. Binding of conjugated antibody was detected using TMB substrate (affymetrix/eBioscience, Santa Clara, USA), and the reaction was stopped by adding 2 N H2SO4. Absorbance at 450 nm, using a 570-nm reference wavelength, was determined with a plate reader (MRX II, Dynex, Chantilly, USA). Cytokine concentrations were calculated by the plate reader software (Revelation, Dynex). Each serum sample was assayed in triplicate.

### Antiinfective therapy

All patients received oral hygiene instructions and professional prophylaxes until the PCR was ≤ 50%. Subgingival debridement (SD) was performed in 2 visits on 2 consecutive days under local anaesthesia (UDS, Sanofi-Aventis Deutschland GmbH, Frankfurt/Main, Germany) according to a modification of the full-mouth disinfection protocol [4, 21]. All teeth exhibiting PPD ≥ 3.5 mm were subgingivally debrided using sonic scalers (Sonicsys, KaVo, Biberach, Germany) and hand instruments. If *Aggregatibacter actinomycetemcomitans* had been detected from subgingival plaque using a commercially available 16S rRNA gene probe test kit (IAI Pado-Test 4.5®, Institut für angewandte Immunologie, Zuchwil, Switzerland) [4], 500 mg amoxicillin and 400 mg metronidazole were prescribed 3 times daily for 7 days. In case of sensitivity to penicillin, 250 mg ciprofloxacin and 500 mg metronidazole were prescribed 2 times daily for 7 days [22–24]. For all patients, oral home care for 14 days after start of SD included the following: rinsing 2 times daily for 60 s with 10 ml 0.12% CHX solution (ParoEx, Schönau, Germany), then brushing of teeth and the back of the tongue with 1% CHX gel. Six and 12 weeks after subgingival debridement, all patients received oral hygiene instructions and professional prophylaxis.

### Statistical analysis

For statistical analysis, a PC programme was used (Systat™ for Windows Version 13, Systat Inc., Evanston, USA). Inferential statistics were intended to be exploratory, not confirmatory. *P* values represent a metric measure of evidence against the respective null hypothesis and were used only to generate new hypotheses. Therefore, neither global nor local significance levels were determined, and no adjustment for multiple testing was applied. *P* values < 0.05 were considered as noticeable. Standard univariate statistical analyses were performed to describe demographic and clinical parameters. Categorical variables are provided as numbers and percentages. Not normally distributed continuous variables are reported as medians (lower/upper quartiles). Patients’ characteristics were compared between BP and CP patients using Fisher’s exact tests for categorical variables and Mann-Whitney *U* tests for non-normally distributed data. The sample size calculation had been done for the main outcome variables NE and CRP for a comparison between ChP and AgP [4].

For all individuals, the Body Mass Index (BMI) and cigarette pack years were calculated. Group frequencies (BP, CP) were expressed for sex and current smoking. Group medians (lower/upper quartiles) were calculated for age, number of remaining teeth, pack years, BMI, GBI, PCR and BOP at baseline and 12 weeks as well as for the changes between baseline and 12 weeks. For all site-based periodontal parameters (PPD, PAL-V, RAL-V), means per individual were calculated at baseline and 12 weeks as well as for changes from baseline to 12 weeks from which group medians (lower/upper quartiles) were calculated. Further, periodontal inflamed surface area (PISA) was calculated per individual to describe the size of the interface between periodontal pocket and vascular system [25].

Putative associations between serum inflammatory markers (CRP, NE, IL-6, Hsp27) and HGB and HCT as well as MCV were screened for by use of simple correlations.

For comparisons, repeated measures analysis of variance (MANOVA) was used for MCV, MCHC and Hsp27 with the following independent variables: time point of examination (baseline, 1 day, 6 and 12 weeks), diagnosis (BP = 0, CP = 1), African origin, sex, age, smoking (never and former smoker = 0, current smoker = 1), adjunctive systemic antibiotics (no = 0, yes = 1), percentage of stage III teeth and bone loss age coefficient. An effect with a probability of *p* < 0.05 was accepted as statistically noticeable.

## Results

Thirty-one ChP and 29 AgP patients were enrolled between October 2006 and December 2009. The results on NE, CRP, leukocyte counts and interleukins 6 and 8, as well as lipopolysaccharide-binding protein had already been published [4]. Three patients were recruited but not enrolled because they did not meet the inclusion criteria. Three patients did not attend the baseline examination. Assignment of new diagnoses according to the 2018 classification was not possible in 3 of the remaining 57 patients because the respective radiographs were not available anymore. Thus, data of 54 patients were analyzed. Of those patients assigned to grade B according to interproximal bone loss in percentage divided by age neither was a current heavy smoker (≥ 10 cigarettes per day) nor suffered from diabetes mellitus. Thus, modifying factors did not change grade. The respective diagnoses after reclassification are provided in Table 1 and patient characteristics are given in Table 2.

**Table 1:** Reclassification of patients

Diagnoses	Chronic periodontitis (*n* = 30)	Aggressive periodontitis (*n* = 24)
Generalized stage III	25	19
Grade B	5	0
Grade C	20	19
Generalized stage IV	5	0
Grade B	3	0
Grade C	2	0
Molar incisor pattern	0	5
Grade C	0	5

**Table 2:** Individuals’ characteristics

Parameters	Periodontitis Grade B (*n* = 8)	Periodontitis Grade C (*n* = 46)	Grade B/C *p*
Female sex: [*n*]/frequency (%)	1 (12.5%)	24 (52.2%)	0.056
Age [years]: median (lower/upper quartile)	62.5 (59.5/65.5)	37 (34/50)	< 0.001
Ethnicity: [*n*]/frequency (%)			
African	0	2 (4.3%)	0.497
Asian	0	5 (10.9%)	
European	8 (100%)	39 (84.8%)	
Remaining teeth [*n*]: median (lower/upper quartile)	26.5 (25/29)	27.5 (26/30)	0.492
Current smokers [*n*]/frequency (%)	1 (12.5%)	15 (31%)	0.411
Former smokers [*n*]/frequency (%)	2 (25%)	9 (20%)	0.659
Pack years: median (lower/upper quartile)	3 (0/19.3)	0.4 (0/12)	0.665
Body Mass Index [kg/m^2^]: median (lower/upper quartile)	24.9 (22.6/26)	26 (23.5/28)	0.324

Red blood cell (RBC) analyses were missing for 3 patients for the 6-week re-examination. Hsp27 serum analysis was also missing for the 6-week re-examination for 3 patients of whom one was the same as had RBC missing. One patient did not attend the 12-week re-examination without giving any reason. RBC analyses were missing for additional 4 patients for the 12-week re-examination. Hsp27 serum analysis was also missing for 1 additional patients for the 12-week re-examination.

Both groups showed significant clinical improvement (BOP, PPD reduction, PAL-V gain, PISA; *p* < 0.05). MCV was noticeably lower in CP than BP at all examinations, HCT only at baseline. For CP, MCHC was noticeably higher 12 weeks after SD than at baseline and 1 day (*p* ≤ 0.005) and Hsp27 increased noticeably at 1 day after SD (*p* < 0.05). With more females in the CP group, repeated measures analysis of variance revealed female sex to be associated with lower MCHC.

Additionally to earlier analyses, PISA was calculated. PISA was significantly smaller in BP than in CP 12 weeks after SD (*p* = 0.046). SD reduced PISA significantly to approximately 1/3 of its baseline size (Table 3).

**Table 3:** Individuals’ periodontal variables and change of periodontal variables after therapy (*PAL-V* clinical vertical attachment level, *RAL-V* relative vertical attachment level)

Parameters Median (lower/upper quartile)		Periodontitis Grade B (*n* = 8)	Periodontitis Grade C (*n* = 46)	Grade B/C *p*
Gingival Bleeding Index [%]	Baseline	16 (10.5/17.5)	12 (5/19)	0.526
	6 weeks	5.5 (2.5/8.5)^a^	2 (1/4)^b^	0.113
	12 weeks	4 (3/8)^a^	5 (2/9)^b, c^	0.981
Plaque Control Record [%]	Baseline	32.5 (18.5/60)	33 (26/41)	0.856
	6 weeks	18 (11/31)^a^	28 (16/42)^a^	0.169
	12 weeks	33 (21/41)	26 (16/32)^a^	0.233
Bleeding on probing [%]	Baseline	49 (39.5/53)	50 (43/60)	0.575
	12 weeks	19 (14.5/27.5)^a^	24.5 (19/33)^b^	0.092
Probing pocket depth (PPD) [mm]	Baseline	3.4 (3.2/3.5)	3.7 (3.2/4.0)	0.242
	12 weeks	2.2 (2.0/2.5)^a^	2.7 (2.4/2.9)^b^	0.006
PPD reduction [mm]		1.2 (1/1.4)	1.0 (0.8/1.3)	0.342
Attachment level [mm] (PAL-V)	Baseline	4.2 (3.6/4.7)	3.1 (2.3/4.5)	0.093
(RAL-V)	Baseline	11 (10.2/11.8)	10.8 (9.8/11.9)	0.715
(RAL-V)	12 weeks	10.5 (9.6/11.3)^a^	10.3 (9.3/11.5)^b^	0.715
Attachment gain [mm] (ΔRAL-V)		0.4 (0.2/0.7)	0.5 (0.2/0.7)	0.789
PISA [mm^2^]	Baseline	1118 (1044/1321)	1299 (939/1597)	0.273
	12 weeks	279 (209/399)^a^	397 (298/566)^b^	0.046

Noticeably different to baseline ^a^(*p* < 0.05); ^b^(*p* < 0.001)

Noticeably different to 6 weeks ^c^(*p* < 0.05)

The study failed to show any statistically noticeable differences between BP and CP with regard to erythrocyte count, HGB, MCH, MCHC (Table 4) and number of platelets (Table 4). Haematocrit was noticeably lower for CP than BP at baseline (*p* < 0.05). MCV was smaller for CP than BP at all time points (*p* < 0.05) (Table 4). For CP, MCHC was statistically noticeably higher 12 weeks after SD than at baseline and 1 day after SD. No such effect was observed for BP (Table 4). Also in CP serum, Hsp27 levels were noticeably increased 1 day after SD compared with baseline, and 6 and 12 weeks after SD (Table 5).

**Table 4:** Individuals’ erythrocyte parameters [median (lower/upper quartile)]

Parameters		Periodontitis Grade B (*n* = 8)	Periodontitis Grade C (*n* = 46)	Grade B/C *p*
Erythrocyte count [10^6^/μl]	Baseline	4.88 (4.54/5.26)	4.7 (4.43/4.98)	0.257
	1 day	4.67 (4.38/4.84)	4.6 (4.43/4.97)	0.913
	6 weeks	4.67 (4.46/4.77)	4.72 (4.41/4.97)	0.770
	12 weeks	4.88 (4.50/4.93)	4.71 (4.42/5.02)	0.942
Haemoglobin value [g/dl] (HGB)	Baseline	15.1 (14.1/15.55)	14.05 (13.4/15.1)	0.068
	1 day	14.4 (13.9/15.5)	14.1 (13.2/15)	0.348
	6 weeks	14.65 (13.9/15.3)	14.05 (13.3/15.1)	0.324
	12 weeks	14.75 (14/15.2)	14.05 (13.2/15)	0.436
Haematocrit [%] (HCT)	Baseline	44.45 (43.05/46.85)	41.7 (39.5/43.5)	0.014
	1 day	43.7 (41.85/45.25)	41.55 (39.7/43.7)	0.088
	6 weeks	43.95 (42.05/44.2)	40.95 (39.2/43.7)	0.125
	12 weeks	43.2 (42.3/46.5)	41.5 (39.1/43.4)	0.105
Mean erythrocyte cell volume [10^−15^ l/cell] (MCV)	Baseline	93.4 (89.8/94.8)	87.95 (85.1/91)	0.045
	1 day	94.5 (91.85/95.45)	88.75 (85.9/92.6)	0.013
	6 weeks	93.55 (92.4/95.4)	88.2 (86.1/91.0)	0.001
	12 weeks	93.4 (90.65/94.45)	87.85 (85.7/91.3)	0.007
Mean corpuscular haemoglobin [pg/cell] (MCH)	Baseline	30.55 (29.75/32.05)	29.65 (29/31.4)	0.151
	1 day	31.35 (30.15/32.25)	30.35 (28.8/31.7)	0.151
	6 weeks	31.1 (30.5/31.85)	30.4 (28.8/31.9)	0.103
	12 weeks	30.45 (30/31.95)	30 (28.9/31.3)	0.268
Mean corpuscular haemoglobin concentration [g/dl] (MCHC)	Baseline	33.25 (33.05/34.1)	33.55 (32.4/35.9)^a^	0.804
	1 day	33.25 (32.5/33.8)	33.6 (32.3/35.8)^a^	0.907
	6 weeks	33.25 (33/33.9)	34.05 (32.9/35.7)	0.682
	12 weeks	33.1 (32.35/34.5)	34.1 (33/36.2)	0.672

Noticeably different to 12 weeks ^a^(*p* ≤ 0.005)

**Table 5:** Platelets and heat shock proteins [median (lower/upper quartile)]

Parameters		Periodontitis Grade B (*n* = 8)	Periodontitis Grade C (*n* = 46)	Grade B/C *p*
Platelets [10^3^/μl] (PLT)	Baseline	205.5 (182/254)	267.5 (215/307)	0.609
	1 day	200.5 (179/244)	250.5 (194/287)	0.759
	6 weeks	207 (181.5/251)	271 (207/308)	0.661
	12 weeks	213.5 (193/258)	268 (214/315)	0.389
Heat shock protein 27 [ng/ml] (Hsp27)	Baseline	5.22 (3.47/9.87)	6.21 (2.96/10.64)^b^	0.961
	1 day	4.74 (2.91/10.45)	7.05 (3.68/12.04)	0.436
	6 weeks	4.68 (3.27/9.96)	6.42 (3.72/12.72)^a^	0.429
	12 weeks	7.22 (4.3/12.13)	5.88 (2.42/11.57)^b^	0.422

Noticeably different to 1 day ^a^(*p* < 0.05); ^b^(*p* ≤ 0.005)

The study failed to detect correlations between serum inflammatory markers (CRP, NE, IL-6, Hsp27) and HGB, HCT and MCV (data not shown).

Repeated measures analysis of variance revealed African origin (*p* = 0.023), age (*p* = 0.009) and percentage of stage III teeth (*p* = 0.005) between subjects to be noticeably associated with MCV. Within subjects (different time points), African origin (*p* < 0.001) and percentage of stage III teeth (*p* = 0.006) were noticeably associated with MCV. Further, between subjects, female sex (*p* = 0.009) and percentage of stage III teeth (*p* = 0.043), and within subjects bone loss to age index (*p* = 0.033) were associated with MCHC.

## Discussion

The aim of this exploratory analysis of data of a previously published study [4] was the assessment of the effect of nonsurgical periodontal therapy on haematological parameters in 8 patients with untreated grade B (BP) and 46 with grade C (CP) periodontitis. Both groups showed significant clinical improvement (*p* < 0.05). MCV was noticeably lower in CP than BP at all examinations, HCT only at baseline. For CP, MCHC was noticeably higher 12 weeks after SD than at baseline and 1 day (*p* ≤ 0.005) and Hsp27 increased noticeably at 1 day after SD (*p* < 0.05). With more females in the CP group, repeated measures analysis of variance revealed female sex to be associated with lower MCHC. Despite MCV, periodontal diagnosis (BP/CP) failed to influence haematological parameters or serum Hsp27. Nonsurgical periodontal therapy increased MCHC 12 weeks and Hsp27 1 day after SD.

Bacteraemia from periodontal pockets and the resulting systemic spill of proinflammatory cytokines cause an acute inflammatory host response [26–28]. The cohort studied in this analysis exhibited significantly higher serum levels of NE and CRP at baseline and 12 weeks after treatment [4]. This significant difference persisted even 5 years after treatment indicating a stronger inflammatory response in AgP than in ChP [5]. One day after scaling, an inflammatory host response was observed in the patients of this study as elevated levels of elastase, CRP, LBP and IL-6 in both ChP and AgP [4]. Does this spill of bacteria and cytokines have an effect on erythrocytes, platelets and Hsp27? And how is the effect of the actual classification on this? This would be expected, based on the ‘anaemia of chronic inflammation’ mediated by the effects of high levels of IL-6 and as a consequence of hepcidin, resulting in higher iron trapping within macrophages and liver cells [13]. Reduced HCT may lead to fatigue and reduced wellbeing. Reduced PLT may influence coagulation and wound healing. Will this effect be different between BP and CP and will it be influenced by treatment?

The parakeratinized and ulcerated pocket epithelium of established gingivitis and periodontitis allows oral microorganisms to enter the underlying tissues and circulation. Combining the pocket walls of all periodontally compromised teeth in an untreated patient, the wound surface, due to periodontitis, is estimated to be as large as 8 to 20 cm^2^ [29]. The size of this wound surface was assessed in this analysis as PISA which ranged in this study from 9 to 15 cm^2^ in BP and from 3 to 25 cm^2^ in CP at baseline.

This study failed to provide any statistically noticeable differences between BP and CP with regard to erythrocyte count, HGB, MCH, MCHC and PLT. However, MCV was noticeably smaller for CP than BP at baseline, 1 day and 6 and 12 weeks after SD (*p* < 0.05). Sex [30, 31] and African origin [30] have an effect on HCT and, thus, on MCV. Hence, repeated measures analysis of variance (MANOVA) controlling for sex and African origin was run to explain the independent variable MCV. The analysis demonstrated statistically noticeable between subject differences due to African origin, age and percentage of stadium III teeth at baseline. More individuals of African origin (*n* = 2 vs. 0) were included in the CP vs. BP group. In univariate comparisons, these differences were not significant. However, multivariate analysis revealed them to explain the differences regarding MCV within the cohort. Repeated measures MANOVA controlling for sex and African origin was also run to explain the independent variable MCHC. The analysis demonstrated statistically noticeable between subject differences due to female sex and percentage of stadium III teeth at baseline. Regarding treatment within subject comparisons revealed a noticeable effect with regard to bone loss age coefficient (*p* = 0.033). Blood haemoglobin concentrations are higher in smokers and heavy alcohol consumers than in non-smokers and abstainers [32]. Although MANOVA included smoking as independent variable, the analysis failed to reveal smoking as a significant factor. This analysis distinguished between current smokers and former/never smokers, whereas Milman and Pedersen compared non-smokers with heavy smokers (> 10 cigarettes per day) [32]. Alcohol intake was not recorded. Compared with Milman and Pedersen who investigated 1437 individuals, this cohort with 54 patients is likely to be too small to show any effect of smoking [32].

Molecular chaperones including Hsp27 increase during cell stress and they may be found elevated in the systemic circulation as a response to inflammation [6]. A previous study found significantly decreased serum Hsp27 in AgP compared with ChP and periodontally healthy controls [9]. In the present study, no differences in Hsp27 levels between BP and CP were found, except an increase in Hsp27 in CP 1 day after treatment. How can this different outcome be explained? Although analyzed by the same laboratory, Hsp27 values of the German cohort were higher than those of the British cohort. The German analysis compares BP and CP, whereas the British study compares ChP with AgP. For the German cohort, clinical severity of periodontitis was provided as mean PPD, PAL-V and PISA [4], whereas, for the British percentage of sites with PPD, > 4 mm is provided [9] These parameters are difficult to compare. It may be that there is a difference in severity between both cohorts. Further, Kaiser et al. chose Hsp27 as the main outcome variable [9], whereas this is an exploratory analysis of a cohort originally aiming at NE and CRP [4]. Both samples are small and, thus, likely to be underpowered.

What are the limitations of this analysis? First of all, this is an exploratory analysis of a cohort originally aiming at NE and CRP [4]. The exploratory analyses were not adjusted for multiple testing. Thus, there is a high risk to detect differences that are due to chance. Further, the sample size is quite small with a high risk to be underpowered. Originally, this study was designed to compare ChP with AgP. After this distinction has been abandoned in the actual classification of periodontal diseases, this is an attempt to use the new diagnoses (BP/CP). However, with no (erythrocytes) or few (Hsp27) studies dealing with this issue with regard to periodontitis, this analysis may raise hypotheses (i.e. neither periodontal diagnosis nor treatment affect erythrocyte parameters).

Within the limitations of the present study, the following conclusion may be drawn: Periodontal diagnosis (BP/CP) fails to influence erythrocyte parameters or serum Hsp27. Nonsurgical periodontal therapy does not seem to influence erythrocyte parameters or Hsp27.
